# Cardiotocography findings of early‐stage chronic fetomaternal hemorrhage after the presentation of reduced fetal movement

**DOI:** 10.1002/ccr3.2057

**Published:** 2019-02-11

**Authors:** Yuria Haruna, Shunji Suzuki

**Affiliations:** ^1^ Department of Obstetrics and Gynecology Japanese Red Cross Katsushika Maternity Hospital Tokyo Japan

**Keywords:** cardiotocography, early‐stage chronic fetomaternal hemorrhage, fetal movements

## Abstract

In the current case of fetomaternal hemorrhage with reduced fetal movements, the findings of cardiotocography (CTG) seemed to be indicating reassuring fetal status; however, a late deceleration and sinusoidal heart rate (SHR)‐like findings were observed following a weak uterine contraction. Altogether, this case indicates that the presence of reduced fetal movements may precede the appearance of SHR patterns on CTG in cases of chronic fetomaternal hemorrhage.

## 1 INTRODUCTION

Fetomaternal hemorrhage refers to the entry of fetal blood into the maternal circulation before or during labor.^1^, ^2^, ^3^ The occurrence of massive fetomaternal hemorrhage is rare; however, the loss of high blood volumes from fetuses may result in various fetal conditions, ranging from cardiovascular distress to death. Thus, the early detection of the symptoms and signs of potential fetomaternal hemorrhage is critical in order to take appropriate action to prevent further harm to the fetus and mother in cases of fetomaternal hemorrhage.^1^ However, the symptoms of fetomaternal hemorrhage are highly nonspecific and include neonatal anemia, followed by decreased or absent fetal movement as well as stillbirth.^1^ Moreover, a standardized management protocol is needed to improve fetal‐neonatal outcomes in such cases.

We present here a case of fetomaternal hemorrhage with emphasis on changes in cardiotocography (CTG) findings indicating the development of chronic fetomaternal hemorrhage.

## 2 CASE PRESENTATION

**Figure 1:**
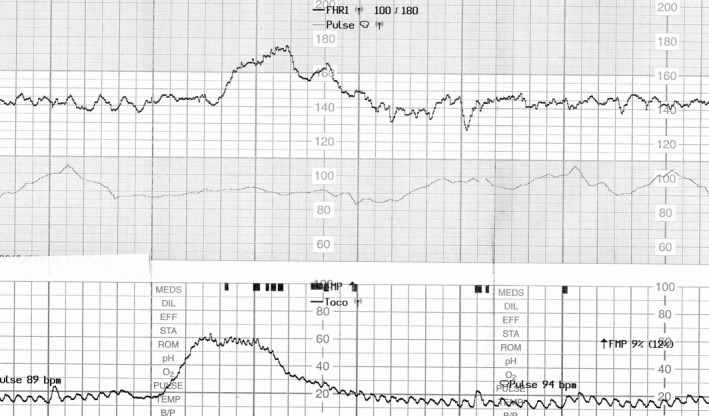
Cardiotocography at 37 wk gestation. An acceleration of 30 beats per minute with normal baseline variability is observed

**Figure 2:**
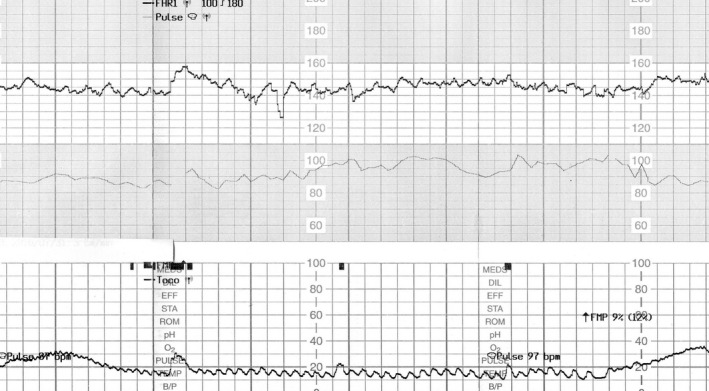
Cardiotocography at 38 wk gestation. An acceleration of 10 beats per minute with normal baseline variability is observed

**Figure 3:**
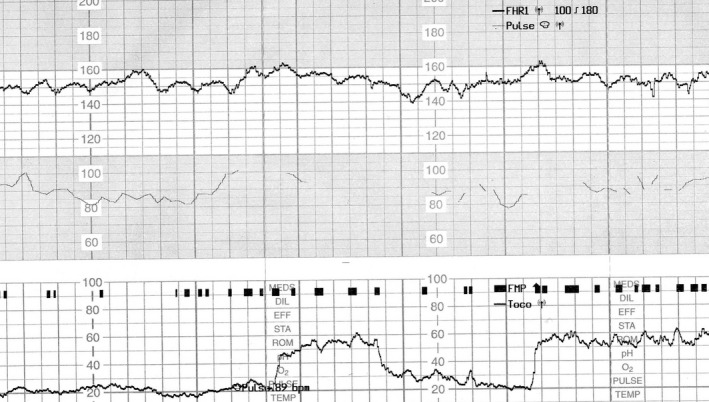
Cardiotocography 4 h after admission at 38 wk gestation. The baseline variability decreased without obvious accelerations

**Figure 4:**
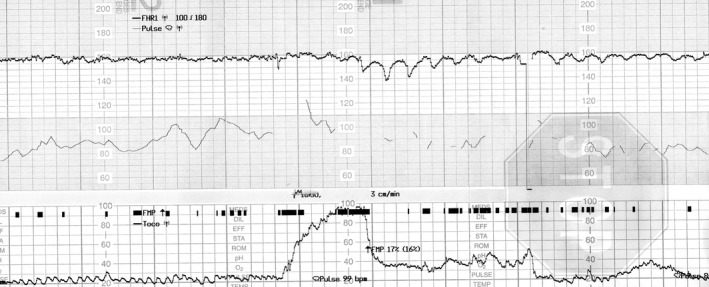
Cardiotocography at 4 h and 40 min after admission at 38 wk gestation. Late deceleration and sinusoidal heart rate‐like findings were observed following a weak uterine contraction

A 30‐year‐old woman (gravida 3 para 1) was admitted to our hospital for elective repeated caesarean delivery at 38 weeks’ gestation. Her pregnancy had progressed uneventfully. Based on the interview at admission, the woman reported of feeling decreased fetal movements from 3 days prior to admission. She also had weak uterine contractions at 1‐2 times per hour. At 37 weeks’ gestation, CTG showed normal baseline findings with normal variability and an acceleration of approximately 30 bpm (Figure 1); however, a diminished acceleration of 10‐15 bpm with normal baseline variability was shown on the CTG at admission (Figure 2). Four hours later, the baseline variability decreased, and the acceleration became unclear on the CTG (Figure 3). Further 40 minutes later, a late deceleration and sinusoidal heart rate‐like findings were observed following weak uterine contraction (Figure 4). Cesarean section was performed, and a 2746‐g pale, female infant was delivered with Apgar scores of 7 and 8 at 1 and 5 minutes, respectively. The umbilical artery pH was 7.344; however, the hemoglobin concentration was 4.2 g/dL (normal: 13‐22 g/dL) with reticulocyte counts of 19.0% (normal: <7%). The maternal hemoglobin‐F and serum alpha‐fetoprotein levels were 4.8% (normal: <1.0%) and 2860 ng/mL (10.2 multiple of median), respectively. Altogether, the case was diagnosed as fetomaternal hemorrhage.

## 3 DISCUSSION

Decreased or absent fetal movement and a sinusoidal heart rate (SHR) pattern have been reported to be useful in diagnosing massive fetomaternal hemorrhage.^1^, ^2^, ^3^ The occurrence of SHR pattern is also well documented in cases with severe fetal anemia.^4^ However, in our earlier case series,^5^ in cases of acute blood loss with normal reticulocyte level, increases in variability have been observed in response to acute hypoxia.^6^ Moreover, acute asphyxia causes stimulation of the autonomic nervous system leading to fetal distress.^7^ Therefore, the presence of SHR pattern may also indicate the late‐staged fetal anemia.

In the current case, the changes in CTG findings indicated the development of chronic fetomaternal hemorrhage. Unfortunately, an ultrasound scan with Doppler, which would have helped us suspect the diagnosis of fetal anemia, was not performed, because the CTG findings at admission seemed to be indication reassuring fetal status.^1^, ^2^, ^3^, ^8^ Moreover, the usefulness of fetal cerebral Doppler in the early stage of fetomaternal hemorrhage with reassuring fetal status indications on the CTG has not been well documented.^8^, ^9^ Therefore, the accumulation of similar cases of progressive fetal hemorrhage is needed to confirm the characteristic changes in CTG findings and their difference from those of fetal hypoxia/anemia.^10^, ^11^

In 2017, we reviewed the CTG findings in 26 cases of fetomaternal hemorrhage with decreased fetal movements in Japan that resulted in adverse neonatal outcomes (neonatal death or cerebral palsy).^9^, ^12^ In 23 of them (88%), a nonreassuring fetal status, such as an adverse SHR pattern, was observed on CTG at the first obstetric consultation conducted based on the mother feeling decreased fetal movements; however, in the remaining 3 cases (12%), a reassuring fetal status was noted on CTG. At their second visit, severe decelerations and/or bradycardia were observed on CTG.

The timing of hospitalization in the current case might be attributable to the deterioration of the fetal condition associated with the development of chronic fetomaternal hemorrhage. If the woman had consulted the hospital 2‐3 days earlier, she might have returned home with the evaluation of fetal well‐being. However, the current case highlights the need to advise mothers to visit the hospital earlier when they feel decreased fetal movement. At admission in the current case, the baseline variability in CTG had been maintained in the absence of fetal distress; however, the hypoxia due to uterine contractions progressively reduced the baseline variability and caused the SHR pattern on CTG. Thus, CTG findings in the current case indicate the development of chronic fetomaternal hemorrhage. Moreover, our case suggests that the presence of reduced fetal movements may precede the appearance of SHR patterns in CTG.

Altogether, it is crucial to listen to the complaints of the mothers and perform a diagnostic procedure for fetomaternal hemorrhage such as CTG and/or ultrasonographic examination. In case of a sustained feeling of decreased fetal movements, like in the current case, continuous CTG or re‐monitoring of CTG should also be considered. In addition, the contraction stress test may be useful for the differential diagnosis of early‐stage chronic fetomaternal hemorrhage with reassuring fetal status on the CTG.

## CONFLICT OF INTEREST

None declared.

## AUTHOR CONTRIBUTION

YH: conceived and designed the report, collected the data, analyzed the data, wrote, and reviewed the manuscript. SS: conceived and designed the report, analyzed the data, wrote and reviewed the manuscript, and approved the final draft.
